# Supplementary material for: The Loss of HJV Aggravates Muscle Atrophy by Promoting the Activation of the TβRII/Smad3 Pathway
Source: Int J Mol Sci. 2025 Feb 26;26(5):2016. doi: 10.3390/ijms26052016 (PMC11900576; doi:10.3390/ijms26052016)
Supplement: Supplementary file 1 [file ijms-26-02016-s001.zip › ijms-3474814-supplementary.pdf]

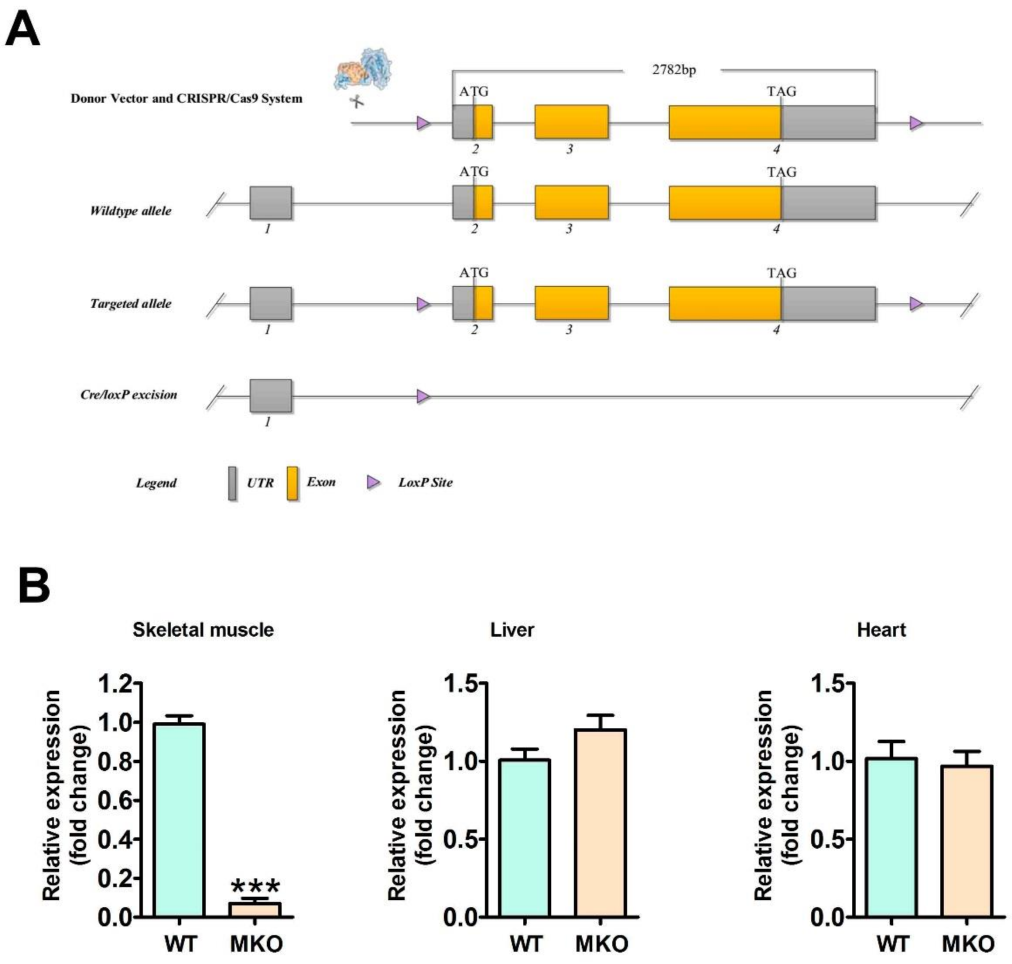


**Supplementary Figure S1. Generation of *HSA-Cre;Hjv*$^{flox/flox}$ mice and phenotype characterization.**

(A) Schematic of the generation of *HSA-Cre;Hjv*$^{flox/flox}$ mice. The HSA-Cre transgene expressed in skeletal muscle was used to recombine the loxP sites flanking Hjv exons 2 to 4 to generate *HSA-Cre;Hjv*$^{flox/flox}$ mice. (B) qRT-PCR analysis of the expression of *Hjv* in the skeletal muscle, liver and heart of *Hjv*$^{flox/flox}$ (WT) and *HSA-Cre;Hjv*$^{flox/flox}$ mice (MKO). Data are shown as means ± SD. ***$P < 0.001$ *vs*. WT mice (*t*-test)

**Supplementary Table S1.** Primers used for genotyping and RT-PCR analysis

| Primers for genotyping | **Sequence** |
|---|---|
| *Hjv loxp* | Forward: 5′-CTTAGCCAGCCAGCGCATTC-3′ |
| | Reverse: 5′-TCCACAAGGTCGGTGGCTTT-3′ |
| *HSA-Cre* | Forward: 5′-GCCTGCATTACCGGTCGATGCAACGA -3′ |
| | Reverse: 5′-GTGGCAGATGGCGCGGCAACACCATT-3′ |
| Primers for RT-PCR | **Sequence** |
| *Hjv* | Forward: 5′-TCACTCCCAGTGCAAGATCC-3′ |
| | Reverse: 5′-ATGCACCGCAGAGTGGAAAG-3′ |
| *Atrogin-1* | Forward: 5′-CTTCAAAGGCCTCACGATCAC-3′ |
| | Reverse: 5′-CAGCCTCTGCATGATGTTCAG-3′ |
| *MuRF1* | Forward: 5′-TTGTGGAGACCGCCATCC-3′ |
| | Reverse: 5′-TCCTTCTTCATTGGTGTTCTTCTT-3′ |
| *18s* | Forward: 5′- CCAGAGCGAAAGCATTTGCCAAGA-3′ |
| | Reverse: 5′- TCGGCATCGTTTATGGTCGGAACT-3′ |
